# STAMP2 Expression Mediated by Cytokines Attenuates Their Growth-Limiting Effects in Prostate Cancer Cells

**DOI:** 10.3390/cancers13071579

**Published:** 2021-03-30

**Authors:** Nicklas Pihlstrøm, Yang Jin, Zeynep Nenseth, Omer F. Kuzu, Fahri Saatcioglu

**Affiliations:** 1 Department of Biosciences, University of Oslo, 0315 Oslo, Norway; pihlipost@gmail.com (N.P.); yang.jin@ibv.uio.no (Y.J.); h.z.nenseth@ibv.uio.no (Z.N.); 2 Institute for Cancer Genetics and Informatics, Oslo University Hospital, 0188 Oslo, Norway

**Keywords:** STAMP2, STEAP4, cytokines, IL-6, IL-1β, prostate cancer, androgen treatment

## Abstract

**Simple Summary:**

Prostate cancer (PCa) is the most common non-skin cancer and one of the leading causes of cancer death in men. Despite significant developments in therapy options with improved survival, no curative treatment is currently available. We have previously identified six transmembrane protein of prostate 2 (STAMP2) as an important factor for PCa growth and survival. We now show that STAMP2 expression is regulated by inflammatory signaling, which has recently been implicated in PCa. Two proinflammatory cytokines, interleukin 6 and interleukin 1 beta, synergize with each other to induce STAMP2 expression. Interestingly, STAMP2 knockdown increased the sensitivity of PCa cells to cytokine treatment. Thus, STAMP2 that acts as a survival factor in PCa, is both independently and synergistically regulated by inflammatory signaling that may affect disease progression.

**Abstract:**

Inflammatory events and dysregulated cytokine expression are implicated in prostate cancer (PCa), but the underlying molecular mechanisms are poorly understood at present. We have previously identified six transmembrane protein of the prostate 2 (STAMP2, also known as STEAP4) as an androgen-regulated gene, as well as a key regulator of PCa growth and survival. STAMP2 is also regulated by, and participates in, inflammatory signaling in other tissues and pathologies. Here, we show that the proinflammatory cytokines interleukin 6 (IL-6) and Interleukin 1 beta (IL-1β) significantly increase and strongly synergize in promoting STAMP2 expression in PCa cells. The two cytokines increase androgen-induced STAMP2 expression, but not expression of other known androgen target genes, suggesting a unique interplay of androgens and cytokines in regulating STAMP2 expression. Interestingly, STAMP2 knockdown significantly increased the ability of IL-6 and IL-1β to inhibit PCa cell growth in vitro. These results suggest that STAMP2 may represent a unique node through which inflammatory events mediate their effects on PCa growth and survival.

## 1. Introduction

Prostate cancer (PCa) is the most common non-cutaneous cancer in men and is a major cause of morbidity and mortality [1]. Androgens play a critical role in PCa progression and form the basis for endocrine therapy that is the standard of care in androgen receptor (AR)-positive PCa [2]. Despite advances in treatment modalities that have improved overall survival, cases detected at more advanced stages often develop into metastatic castration-resistant PCa (mCRPC), for which there is currently no available curative treatment [3].

The prostate gland is frequently subject to dietary, physical, and microbiological insults that can give rise to inflammation [4]. Chronic intraprostatic inflammation is suspected to play a mechanistic role in the pathogenesis of PCa and constitutes a risk factor along with age, obesity, and hereditary factors [4,5]. Treatment-naïve PCa patients initially present with a diverse degree of immune infiltration, from immunologically “cold” primary tumors to cases with heavy infiltration of cells from both the innate and adaptive arms of the immune system [6,7], but features of chronic inflammation are found in higher proportions of cases of both prostatic intraepithelial neoplasms (PIN) and PCa [8].

Intraprostatic inflammation includes recruitment and pseudo-differentiation of immature myeloid cells, e.g., myeloid-derived suppressor cells (MDSCs), in addition to other cell types [4,9]. Innate immune cells of the myeloid lineage are the principal sources of the inflammatory cytokines tumor necrosis factor (TNF), interleukin 1 beta (IL-1β), and interleukin 6 (IL-6) in the tumor microenvironment (TME) [10]. The levels of both IL-6 and its receptor (IL-6Rα) are elevated in localized PCa samples compared to the adjacent healthy tissue and are correlated with increased proliferation measured by Ki67 expression [11,12]. Elevated circulating IL-6 level is a prognostic factor for poor prognosis in PCa patients [13,14]. Furthermore, signal transducer and activator of transcription 3 (STAT3), the transcription factor that is hyperactivated by upstream IL-6/IL-6Rα/Janus kinase (JAK) signaling cascades in many tumors with features of chronic inflammation, has been implicated in maintaining a PCa stem-like cell type that can survive in an androgen receptor (AR)-independent manner [15,16]. 

Immunohistochemistry (IHC) analysis of localized PCa tumors from 118 neoadjuvant-naïve patients who underwent radical prostatectomy showed great variability in the expression levels of inflammatory mediators among tumors [17]; however, the expression of IL-6, IL-1β, and nuclear factor (NF) kappa B (NF-κB), a known mediator of inflammatory responses, were detected in almost all samples (>94–95%). In addition, high IHC scores for IL-1β were associated with earlier biochemical recurrence (BCR) [17,18]. These studies indicate that inflammatory events and dysregulated cytokine expression are frequent features of PCa, which might be involved in pathogenesis and disease progression.

A protein that can potentially influence inflammatory pathways in PCa is six transmembrane protein of the prostate 2 (STAMP2, also called six transmembrane epithelial antigen of prostate 4—STEAP4). We initially identified STAMP2 as an androgen-regulated gene in PCa cells [19]. STAMP2 is a metalloreductase [20,21,22] that is highly expressed in the normal prostate and has a key role in PCa proliferation [23]; in addition, it may also regulate inflammatory homeostasis in a context-dependent manner in different tissues, such as adipose tissue, macrophages, inflammatory skin conditions (psoriasis), and in rheumatoid arthritis (RA) [24,25,26,27].

Suggesting a role in inflammatory responses, both IL-6 and IL-1β induced STAMP2 expression in 3T3-L1 murine adipocytes [28]. Furthermore, STAMP2 expression is elevated in myeloid cells from human RA patients and in murine models of RA, where its expression was reduced by the TNF blockade [26,27,29]. The function of STAMP2 in these contexts appears to result in a downregulation of inflammation through reduced cytokine and chemokine synthesis, and thereby in the migration and activity of immune cells [27,30].

Given that inflammation is implicated in PCa while STAMP2 expression and function are both linked to inflammatory signaling in other cellular contexts, we investigated the potential regulation of STAMP2 by inflammatory stimuli in the PCa cell lines LNCaP and C4-2B. Here, we report that inflammatory cytokines IL-6 and IL-1β induce *STAMP2* expression both alone as well as in a synergistic manner. Mechanistically, this induction is independent of the AR and relies on STAT3 and NF-κB signaling in low-hormone culture conditions. We further show that *STAMP2* expression responds positively and synergistically to simultaneous IL-6/STAT3 and androgen/AR signaling in C4-2B cells as opposed to canonical target genes of either pathway. Finally, we show that the presence of STAMP2 promotes PCa cell survival in inflammatory conditions over time.

## 2. Materials and Methods

### 2.1. Cell Culture and Treatments

Human PCa cell lines LNCaP, VCaP, C4-2B, and 22Rv1 were purchased from the American Type Culture Collection (Rockville, MD, USA). The cells were routinely kept in a humidified 5% CO_2_ and 95% air incubator at 37 °C. The PCa cells were cultured in the RPMI 1640 medium containing 10% fetal bovine serum (FBS), 50 U/mL penicillin, 50 µg/mL streptomycin, and 4 mM l-glutamine (Lonza, Basel, Switzerland). All cell lines were routinely tested and were free of mycoplasma contamination. 

For experiments analyzing responses to androgens, the cells were maintained in the RPMI medium supplemented with 5% charcoal-treated (CT) FBS for at least two days before replacing the medium with 1% CT-FBS + RPMI 1640 with or without 1 nM of synthetic androgen R1881 (Sigma-Aldrich, St. Louis, MO, USA) for 24 h (or other period as indicated). The control cells were treated with 0.01% ethanol in the same medium (vehicle controls). 

Recombinant TNFα, IL-1β, and IL-6 (GenScript, Piscataway, NJ, USA) were diluted in PBS. As 20 ng/mL of both IL-1β and IL-6 consistently gave robust induction of STAMP2 expression, this was the concentration used unless otherwise noted. In combinatorial treatment experiments, 20 ng/mL of each cytokine were used.

For cytokine induction, the cells were seeded in a regular growth medium and cultured until 80–90% confluence. In short-term induction experiments, the cells were pretreated with serum-free RPMI for 2–5 h prior to dropwise induction with cytokines diluted in serum-free RPMI. In long-term experiments, the cells were grown in 2% FBS media supplemented with cytokines. An equal volume of PBS was added to the medium of the control cells.

### 2.2. RNA Interference

Small interfering RNAs (siRNAs) used to silence *STAMP2*, *STAT3*, *RELA*, and *AR* were purchased from Qiagen. Allstar Negative Control siRNA (Qiagen, Hilden, Germany) was used as a negative control. The cells were reverse transfected with 5 nM siRNA per well/plate using Lipofectamine RNAiMAX (Invitrogen, Carlsbad, CA, USA) and diluted in RPMI media without supplements. When targeting *STAMP2* or *STAT3*, two different siRNAs for each gene were pooled together and used at a final concentration of 2.5 nM each. The medium was changed the next day and the cells were cultured further in an antibiotic-free 10% FBS RPMI medium for 1–2 days before replacing the medium with the RPMI medium supplemented with 2% FBS and cultured overnight prior to dropwise cytokine treatment the following morning. Small interfering RNA sequences are available upon request.

### 2.3. Quantitative PCR

RNA extraction, cDNA synthesis, and quantitative PCR were performed as described previously [31]. Briefly, cultured cells were washed with ice-cold PBS before RNA extraction. RNA was extracted using the TRI reagent (ThermoFisher Scientific, Waltham, MA, USA) according to the manufacturer’s instructions. RNA concentration and purity were determined spectroscopically using Nanodrop 2000 (ThermoFisher Scientific) and RNA integrity was confirmed by agarose gel electrophoresis. For cDNA synthesis using a standard SuperScript II/IV reverse transcriptase (ThermoFisher Scientific), 1 µg total RNA from each sample was used. Quantitative PCR was performed using the cDNA template at a final concentration corresponding to a 1:50 dilution of RT reactions, gene-specific primers at a final concentration of 0.5 µM each, and the 2× SYBR Green master mix reagent (Bimake, Munich, Germany) in 10 µL reactions. Quantitative PCR reactions were run and analyzed using a STEPONE plus system (R&D Biosystems, Minneapolis, MN, USA). *RPLP0* and/or *ACTB* expression was analyzed alongside each run with genes of interest and served as internal reference genes. The data shown are representative of at least two independent experiments performed in triplicate except for time–course experiments. Primer sequences are available upon request.

### 2.4. Western Blot Analysis

Whole-cell extracts and Western blot analyses were performed using standard methods as described previously [32]. Nuclear fractions were prepared using a Subcellular Protein Fractionation Kit for Cultured Cells (ThermoFisher Scientific). The following antisera were used: from Cell Signaling Technology, STAT3 (#9145), phospho-STAT3 (#9145), phospho-STAT1(#14594), p38 (#9212), phospho-p38 (#9211), p42/44 (#9102), phoshpho-p42/44 (#9101), JNK (#9252), and phospho-JNK (#9251); from Santa Cruz Biotechnology, AR (sc-816), phosphor c-Jun (sc-16312), phospho-p65, IκB (sc-371), GAPDH (sc-47274), and β-actin (sc-47778); from Proteintech (Manchester, UK), HDAC1 (10971). Unfortunately, we were not able to either procure or produce a reliable antibody against STAMP2, and therefore we could not study the dynamics of STAMP2 expression at the protein level.

### 2.5. Chromatin Immunoprecipitation (ChIP)

The ChIP experiment was carried out according to the standard protocol (Upstate Biotechnology, New York, NY, USA) as described previously [32]. LNCaP cells were plated in 15-cm tissue culture plates and cultured as described above. For α-AR ChIP, the cells were treated with 1 nM R1881 or vehicle for 24 h followed by a crosslinking step (1% formaldehyde at 37 °C) and a quenching step with 125 mM glycine. Chromatin was fragmented using a Bioruptor sonicator (Diagenode, Liège, Belgium) and was immunoprecipitated with antibodies against AR or IgG (Vector Laboratories; 1:1000). After reversal of crosslinking, immunoprecipitated DNA, as well as input DNA, was quantified by qPCR. PCR primers used are available upon request. Standard curves were created by 10-fold serial dilutions of an input template. 

### 2.6. Small-Molecule Inhibitors

Stock solutions of the small-molecule inhibitors targeting AR (MDV3100, Selleckchem, Houston, TX, USA), JAK2 (AZD1480, Selleckchem), MEK2 (PD98059, Calbiochem, San Diego, CA, USA), JNK (SP600125, Calbiochem), or p38 mitogen-activated protein kinases (MAPKs) (SB203580, Calbiochem) were prepared as 1000× stock solutions in DMSO which were then diluted in serum-free RPMI to treat the cells. A solution of 0.1% DMSO diluted in RPMI served as the vehicle control. The medium was replaced with the inhibitors for 2–4 h prior to cytokine induction as described above.

### 2.7. Cell Viability and Colony Formation Assay

The cells were plated in 96-well plates and treated as indicated elsewhere. Cell viability was determined using the tetrazolium salt CCK-8 assay (Bimake). For the colony formation assay, the transfected cells were treated with IL-1β, IL-6, or both and cultured for 10 days. The cells were then rinsed with ice-cold PBS, subjected to the CCK-8 assay, fixed with methanol, and stained with 0.4% crystal violet. The colonies were quantified by extracting crystal violet in 10% acetic acid and measuring the absorbance at 590 nm. 

### 2.8. Statistical Analyzes

The means and standard deviations were calculated using Microsoft Excel. The statistical significance was evaluated using a two-sided Student’s *t*-test. Values of *p* < 0.05 were considered significant.

## 3. Results

### 3.1. STAMP2 Is a Direct AR Target Gene in Prostate Cancer Cells

**Figure 1:**
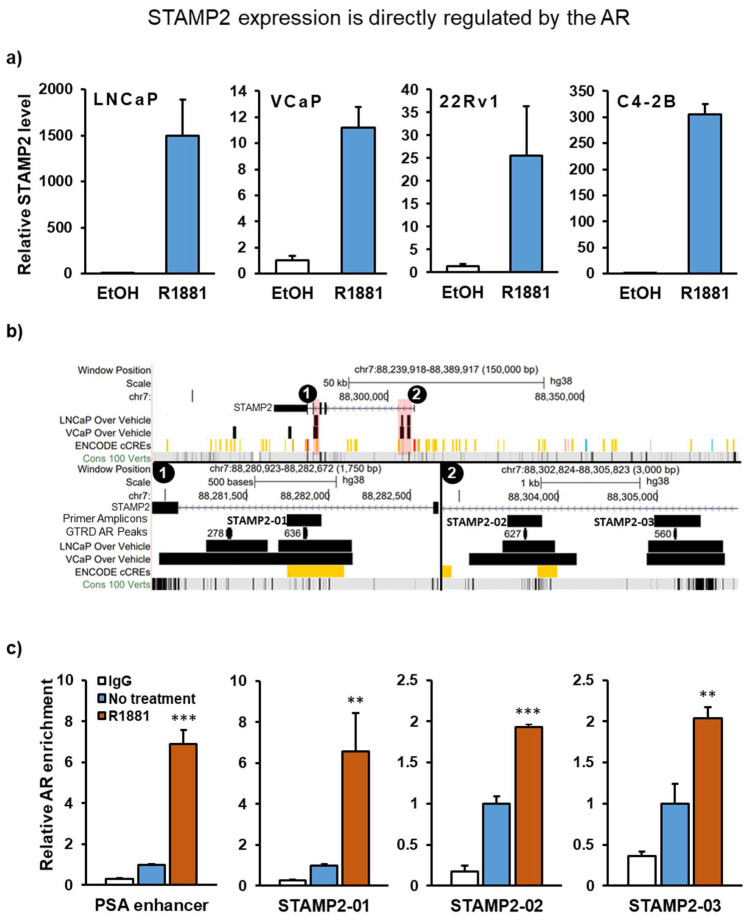
STAMP2 expression is directly regulated by the AR. (**a**) LNCaP, VCaP, 22Rv1, and C4-2B cell lines were androgen-starved for 24 h followed by 1 nM R1881 induction for 24 h. Total RNA was then harvested and subjected to qRT-PCR analysis. (**b**) Putative AR-binding sites in the vicinity of the STAMP2 gene are shown. The upper panel shows AR-binding peaks identified in the LNCaP and VCaP cells in the GSE28126 dataset. The red highlighted binding sites are zoomed in the lower panel as 1 and 2. The numbers in the Gene Transcription Regulation Database (GTRD) lane show the number of AR peak calls in PCa AR ChIP-Seq studies collected in the GTRD (version 20.06). The thicker portion in the peaks shows the summits ± 10 base pairs. The ENCODE cCRE lane shows candidate cis-regulatory elements reported in the Encyclopedia of DNA Elements database. (**c**) LNCaP cells were cultured and treated with the vehicle or R1881 for 24 h. The cells were fixed, and the ChIP assay was performed as described in the Materials and Methods using a mixture of AR antisera. PSA: Prostate-specific antigen, R1881: synthetic androgen methyltrienolone. Statistics indicated with asterisks are compared to no treatment. ** *p* < 0.01, *** *p* < 0.001.

We originally identified *STAMP2* in a screen for androgen-regulated genes that are differentially expressed in PCa [19]. Consistently, *STAMP2* expression is induced by the synthetic androgen R1881 in the AR-positive PCa cell lines LNCaP, VCaP, 22Rv1, and C4-2B (Figure 1a). The dramatic induction in LNCaP cells (around 1500-fold) reflects the very low basal level of *STAMP2* expression in these cells compared to other PCa cells we tested.

STAMP2 has five exons and four introns, where the first intron is unusually long (~22.5 kb, Figure 1b). Previous AR chromatin immunoprecipitation sequencing (ChIP-Seq) experiments in LNCaP and VCaP [33] cells identified two putative AR-binding sites around 1.6 kb apart within intron 1 of *STAMP2* and two adjacent putative sites in intron 3 (Figure 1b—“LNCaP” and “VCaP over vehicle” lanes). AR enrichment on these sites has also been identified in several other experiments, as the Gene Transcription Regulation Database (GTRD) showed a large number of overlapping AR peak calls in these regions (Figure 1b—“GTRD” lane). Moreover, one of the two adjacent putative sites in intron 3 was overlapping with a distal enhancer-like signature according to the ENCODE Candidate Cis-Regulatory Elements database (Figure 1b—“ENCODE” lane). To experimentally validate these sites and investigate their role in androgen-regulated *STAMP2* expression, we performed anti-AR ChIP–quantitative PCR (ChIP-qPCR) experiments after R1881 treatment of LNCaP cells using primer sets targeting the combined putative AR-binding sites in exon 3 and the two independent sites in intron 1 (Figure 1b—“Amplicon” lane). As a positive control, we measured AR binding to a previously characterized AR response element in the *KLK3* enhancer [34]. Androgen treatment resulted in a similar level of enrichment of AR binding to the putative site within intron 3 of *STAMP2* and the one in the KLK3 enhancer (Figure 1c). AR association with each of the two putative sites within intron 1 was also significantly increased upon R1881 treatment, albeit to a lesser extent (2-fold vs. 7-fold) than with the intron 3 site. Enrichment of AR on these binding sites as a result of androgen treatment concurrent with strong induction of mRNA synthesis suggests that *STAMP2* expression is directly regulated by AR after its activation by androgens.

### 3.2. Temporal Regulation of STAMP2 Expression by Cytokines

**Figure 2:**
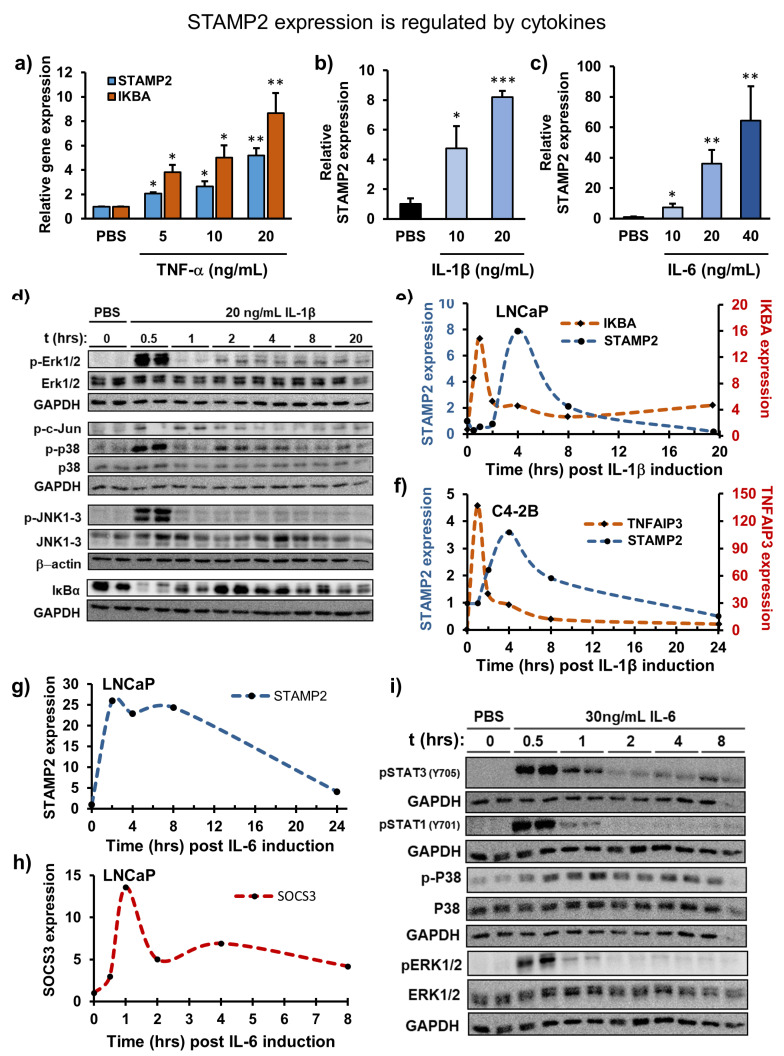
*STAMP2* expression is regulated by cytokines. (**a**–**c**) LNCaP cells were serum-starved and then induced with different concentrations of TNF (**a**), IL-1β (**b**), or IL-6 (**c**) for 16 h. Total RNA was then harvested and subjected to qRT-PCR analysis. Statistics shown by asterisks are relative to PBS treatments. (**d**) LNCaP cells were serum-starved before induction with 20 ng/mL IL-1β. Whole-cell lysates were harvested at the indicated timepoints and subjected to Western blot analysis. (**e**–**h**) LNCaP or C4-2B cells were serum-starved before induction with 20 ng/mL IL-1β (**e**,**f**) or IL-6 (**g**,**h**). Total RNA was harvested at the indicated timepoints and subjected to qRT-PCR analysis. (**i**) LNCaP cells were serum-starved before induction with 30 ng/mL IL-6. Whole-cell lysates were harvested at the indicated timepoints and subjected to Western analysis. pSTAT3, pP38, and p38 blots were developed from the same membrane, and share the same GAPDH as the endogenous control. Similarly, pSTAT1 pERK1/2 and ERK1/2 were developed from the same membrane and hence share the same GAPDH. Asterisks above the bar graphs indicate statistics compared to PBS treatments. * *p* < 0.05, ** *p* < 0.01, *** *p* < 0.001.

In addition to the regulation by androgens/AR, previous work has shown that STAMP2 expression is positively regulated by inflammatory cytokines IL-1β, IL-6, and TNF in various cell types and model systems; however, it is not known whether these factors regulate STAMP2 expression in PCa cells. To determine this, we treated LNCaP cells and their androgen-independent derivative CRPC model cell line C4-2B with TNF, IL-1β, and IL-6 and assessed changes in *STAMP2* expression. STAMP2 mRNA expression was significantly induced by all three cytokines in a dose-dependent manner (Figure 2a–c). Contrary to a previous report of negative results [35], TNF treatment of LNCaP cells led to a modest, but significant induction of *STAMP2*, although weaker if compared to the TNF-induced canonical NF-κB target gene *IKBA* (inhibitor of kappa B alpha) (Figure 2a). At the same concentration, TNF did not induce *STAMP2* expression in C4-2B cells (Figure S1a). In contrast, IL-1β, another upstream activator of NF-κB signaling, led to 7–8-fold induction of *STAMP2* expression in LNCaP cells (Figure 2b), whereas IL-6 treatment led to approximately 80-fold induction (Figure 2c). These data suggest that inflammatory cytokines can drive STAMP2 expression in PCa cells. IL-6 treatment in LNCaP cells resulted in a different *STAMP2* expression profile (Figure 2g,h). IL-6 treatment activated STAT1 and STAT3 along with p38 and ERK MAPKs in LNCaP cells, reaching maximum levels within 30 min (Figure 2i). In contrast, STAMP2 mRNA levels peaked at two hours post-IL-6 induction and then remained elevated for 6 h before decreasing towards basal levels by 24 h in LNCaP cells (Figure 2g). In contrast, IL-6-induced *STAMP2* expression remained at maximum levels at 24 h in C4-2B cells (Figure S1b). This was in contrast to the expression profiles of the canonical STAT3 target genes SOCS3 and FOS, whose mRNA levels peaked at 1 h and declined to lower levels in 2–4 h (Figure S1c,d).

### 3.3. Cytokines and Androgens Synergistically Induce STAMP2 Expression in PCa Cells

**Figure 3:**
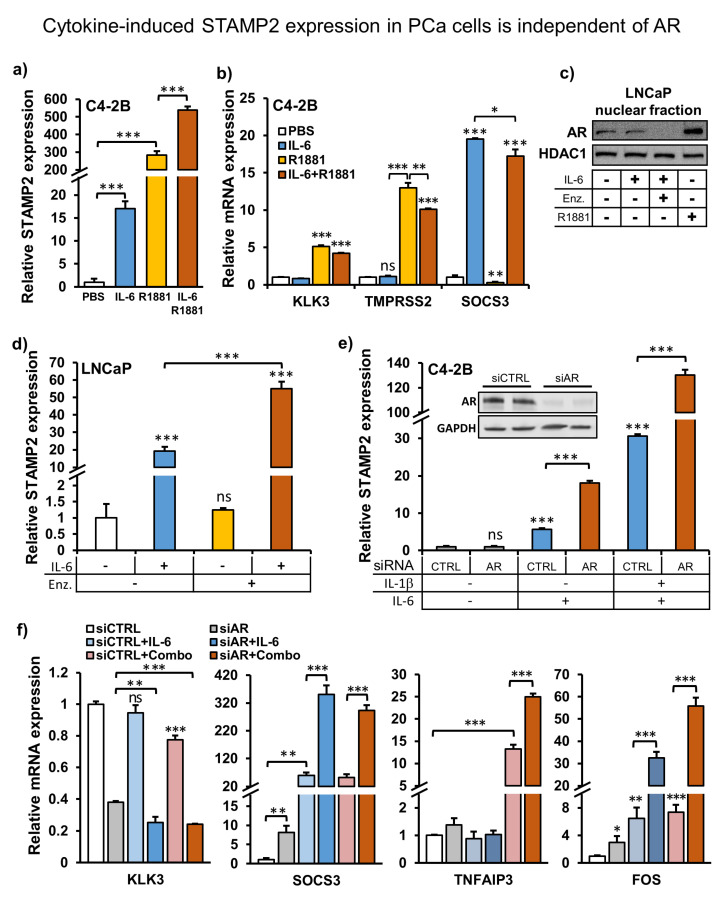
Cytokine-induced STAMP2 expression in PCa cells is independent of AR. (**a**,**b**) C4-2B cells were androgen-starved for 24 h followed by treatment with 20 ng/mL IL-6, 1 nM R1881, or their combination for 24 h. Total RNA was harvested and subjected to qRT-PCR analysis. Statistics shown by asterisks above the columns are relative to respective PBS treatments. (**c**,**d**) LNCaP cells were pretreated with or without 2 μM enzalutamide (Enz.) for 2 h and then treated with 30 ng/mL IL-6 for 2 h. As a control for AR induction, 5 nM R1881 treatment was applied overnight. The cells were harvested for Western blot analysis of the nuclear AR protein level (**c**) and qRT-PCR analysis of STAMP2 expression (**d**). (**e**,**f**) C4-2B cells were cultured and transfected with AR siRNA or control siRNA for 48 h. The cells were then harvested followed by Western blotting for detection of AR protein levels and qRT-PCR for detection of indicated genes expression. Combo stands for combinatorial treatment with IL-6 and IL-1β. Statistics shown by asterisks above the columns are relative to non-induced siCTRL treatment. * *p* < 0.05, ** *p* < 0.01, *** *p* < 0.001.

As we showed that STAMP2 is an AR target gene (Figure 1), we investigated whether the IL-6-induced *STAMP2* expression is mediated through STAT3-driven transactivation of AR [36]. To that end, we cultured C4-2B cells in a steroid hormone-depleted medium for two days before treatment with R1881, IL-6, or both for 24 h. Either treatment alone increased *STAMP2* expression; however, compared to the response to R1881, IL-6-mediated *STAMP2* expression was modest (Figure 3a). Interestingly, the combinatorial treatment led to an almost double *STAMP2* expression relative to the R1881 treatment alone. Enhanced *STAMP2* expression by combinatorial IL-6 + R1881 treatment could result from enhanced AR activation by IL-6. If this is the case, the combinatorial treatment should also increase the expression of other AR target genes. Interestingly, unlike *STAMP2*, expression of canonical AR target genes, such as *KLK3* and *TMPRSS2*, was slightly decreased upon combinatorial treatment compared to androgen induction alone (Figure 3b). Furthermore, R1881 reduced *SOCS3* expression, a STAT3 target gene, both alone when compared to the basal expression and in combination when compared with IL-6 treatment alone (Figure 3b). These data show that *STAMP2* expression is simultaneously induced by both androgens and IL-6 as opposed to canonical target genes of either pathway.

### 3.4. Cytokine-Induced STAMP2 Expression in PCa Cells Is Independent of AR

The data presented above clearly show that cytokines can induce STAMP2 expression, but did not explore whether AR activity is required for this process. To evaluate this, we treated LNCaP cells with the AR antagonist enzalutamide (MDV3100) prior to IL-6 treatment and evaluated *STAMP2* expression. Pretreatment with enzalutamide for two hours efficiently depleted nuclear accumulation and, thus, gene regulation activity of AR in LNCaP cells without altering basal STAMP2 levels (Figure 3c,d). Surprisingly, depletion of nuclear AR did not inhibit STAMP2 expression induced by IL-6; on the contrary, enzalutamide-pretreated cells exhibited higher sensitivity to IL-6 treatment, evidenced by approximately 2.5-fold enhanced *STAMP2* expression (Figure 3d) compared to DMSO-treated control cells. In contrast, expression of the canonical AR target gene KLK3 was inhibited by both treatments, as well as by their combinations (Figure S2a).

Consistently, siRNA-mediated AR knockdown in C4-2B cells led to dramatic increases in cytokine-mediated induction of *STAMP2* expression (Figure 3e). Similar to the enzalutamide-cytokine cotreatment experiment, KLK3 levels were downregulated by both AR knockdown and cytokine treatment (Figure 3f). Expression of STAT3 target genes, SOCS3 and FOS, and the NF-κB target gene, TNFAIP3, followed a similar pattern with STAMP2 expression. Their levels were also dramatically upregulated in AR knockdown cells upon cytokine treatment (Figure 3f). Furthermore, in keeping with previous reports [37], AR protein levels were reduced after IL-1β treatment but were induced by IL-6 treatment (Figure S2b). Independent of this, however, endogenous expression of canonical AR target genes in similar treatment regimens are consistently decreased upon treatment with IL-1β or IL-6 in both LNCaP and C4-2B cells (Figure S2c,d). These data strongly indicate that cytokine-induced expression of *STAMP2* in PCa cells is independent of AR activation; in fact, the androgen/AR axis appears to have inhibitory effects on cytokine-induced *STAMP2* expression.

### 3.5. IL-1β and IL-6 Synergize to Increase STAMP2 Expression

**Figure 4:**
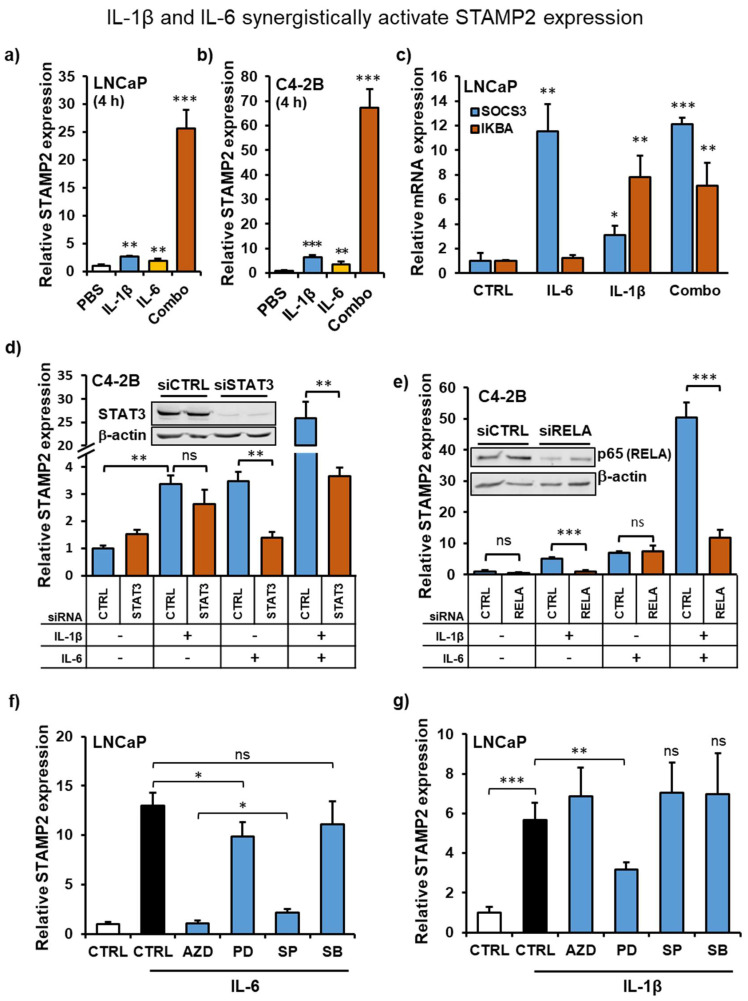
IL-1β and IL-6 synergistically activate STAMP2 expression. (**a**–**c**) LNCaP cells (**a**,**c**) and C4-2B cells (**b**) were serum-starved and then induced with IL-6, IL-1β, or their combination for 16 h. Total RNA was harvested and expression of the indicated genes was determined by qRT-PCR analysis. Statistics shown by asterisks above the columns are relative to respective PBS or CTRL treatments. (**d**,**e**) C4-2B cells were transfected with siRNAs against STAT3 (**d**) or p65 (RELA) (**e**) for 48 h, followed by treatment with IL-6, IL-1β, or their combination for 16 h. The cells were harvested for qRT-PCR and Western blot analysis. The insets show the efficiency of knockdowns. (**f**,**g**) LNCaP cells were pretreated with indicated kinase inhibitors (0.5 µM AZD1480 (AZD), 50 µM PD98059 (PD), 10 µM SP600125 (SP), or 20 µM SB203580 (SB)) in a serum-free medium for 2 h prior to induction with 30 ng/mL IL-6 (for 2.5 h) (**f**) or 20 ng/mL IL-1β (for 4 h) (**g**). Total RNA was harvested and subjected to qRT-PCR analysis. Statistics shown by asterisks above the columns are relative to the control treatments. * *p* < 0.05, ** *p* < 0.01, *** *p* < 0.001.

The proinflammatory cytokines IL-1β and IL-6 are often coexpressed, regulate each other in a paracrine fashion, and synergistically act on target cells or organs [38,39,40]. To assess whether they also synergistically regulate *STAMP2* levels, we treated PCa cells with the two cytokines either alone or in combination and determined STAMP2 expression. Both in LNCaP and C4-2B cells, in contrast to single treatments, cotreatment with the two cytokines led to a dramatically increased induction of STAMP2 expression (Figure 4a,b,d,e). In contrast, expression of SOCS3 or IKBA, target genes of STAT3 and NF-κB pathways, respectively, were not affected by combinatorial treatment (Figure 4c). 

As IL-6 activates STAT1/3 (Figure 2g), IL-1β activates NF-κB (Figure 2b), and both cytokines activate MAPK signaling, we considered these pathways as putative positive regulators of *STAMP2* expression. To investigate this, we used siRNA-mediated knockdown of STAT3 and the NF-κB subunit RelA/p65. As expected, knockdown of STAT3 significantly reduced IL-6, but not IL1-β-mediated *STAMP2* induction (Figure 4d). The strong synergy between IL-6 and IL-1β treatments required STAT3 activity, as its knockdown effectively inhibited *STAMP2* levels to a comparable level to IL-1β treatment alone (Figure 4d). In contrast, *STAMP2* induction mediated by IL-1β, but not IL-6, was dependent on NF-kB signaling through p65 (Figure 4e). Similar to STAT3 knockdown, the synergy between IL-6 and IL-1β was also effectively blocked by RELA knockdown (Figure 4e).

We also assessed the mechanism of cytokine-mediated *STAMP2* expression by perturbing JAK/STAT, MEK/ERK, JNK1/3, and p38 MAPK cascades through pretreatment with pathway-specific small-molecule inhibitors. Consistent with the results using RNAi, pretreatment of LNCaP cells with AZD1480, an ATP-competitive inhibitor of JAK2 which inhibits downstream activation of STAT1/3, effectively blocked the IL-6-mediated induction of *STAMP2* (Figure 4f). AZD1480 pretreatment had no effect on IL-1β-mediated induction of STAMP2 (Figure 4g). Similarly, SP600125, an ATP-competitive inhibitor of JNK1-3, effectively blocked IL-6, but not IL-1β-mediated induction of *STAMP2* expression. The effects of these small molecules were also investigated by Western blotting (Figure S3a,b). The JAK2 inhibitor AZD1480 effectively blocked IL-6 mediated STAT1/3 tyrosine phosphorylation. It also showed efficacy through inhibition of ERK1/2 activation, as the IL-6/IL-6R/Jak axis also branches out to activate Ras/MAPK signaling downstream of Jak transactivation by the receptor. The activity of JNK inhibitor SP600125 was validated through inhibition of IL-1β-mediated c-Jun phosphorylation. However, SP600125 also effectively blocked STAT3 and ERK1/2 activation downstream of IL-6 (Figure S3a). In fact, its effect on STAT3 phosphorylation was observable even at low doses that do not affect c-JUN phosphorylation (Figure S3b). Hence, the effect of it on STAMP2 regulation is likely to be through unintended STAT1/3 inhibition.

On the other hand, inhibition of MEK1/2 and thereby Erk1/2 activation using PD98059 modestly, but significantly reduced STAMP2 induction by both cytokines, whereas p38 MAP kinase inhibitor SB203580 failed to show any effect on cytokine-mediated STAMP2 expression (Figure 4f,g). PD98059 was very effective in inhibiting IL-6 or IL-1β-mediated ERK1/2 phosphorylation, whereas SB203580 failed to show any inhibitory effect on ERK1/2 or STAT3 phosphorylation (Figure S3a). These results show that multiple signaling cascades play a role in cytokine-mediated *STAMP2* expression in PCa cells. 

### 3.6. STAMP2 Expression Attenuates Cytokine-Induced Growth Inhibition in PCa Cells

**Figure 5:**
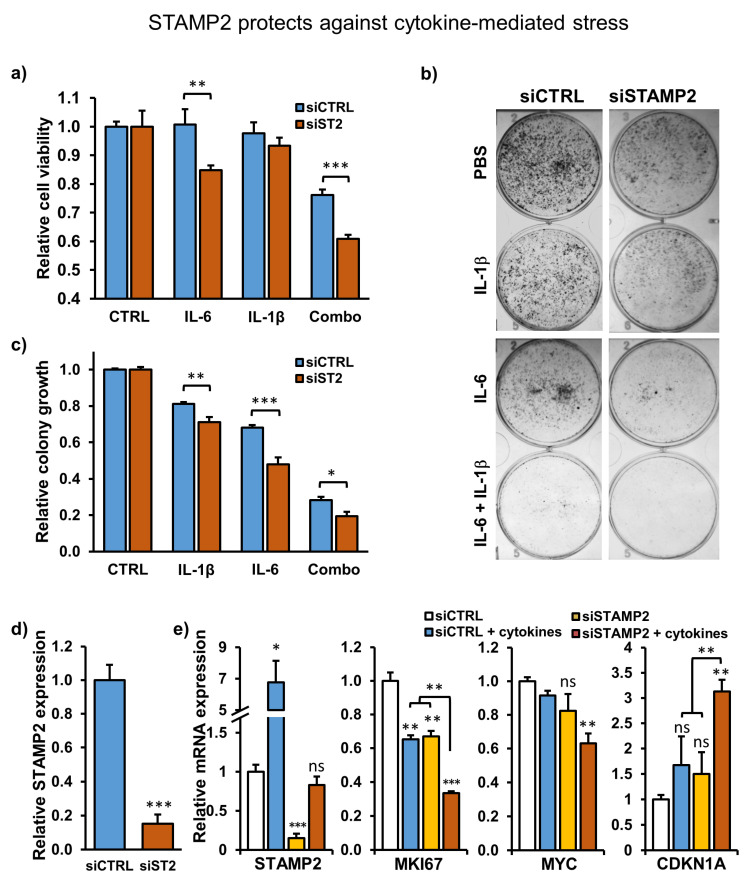
STAMP2 protects against cytokine-mediated stress. (**a**–**c**) C4-2B cells were reverse transfected with 5 nM STAMP2 or control siRNA in a six-well plate and 24 h later harvested and replated into 96-well plates (for cell viability assay) or six-well plates (for colony formation assay). Three hours later, the cells were induced with the indicated cytokines and the viability of the cells was determined 72 h later by the CCK-8 assay (**a**). Colony formation assays were developed nine days post-reseeding (**b**). The relative amount of viable cells in the growing colonies was determined by the CCK-8 assay (**c**) and the efficacy of STAMP2 knockdown was verified by qRT-PCR (**d**). (**e**) The effects of combined (IL-1β and IL-6) treatment on STAMP2 knockdown and control C4-2B cells were determined by qRT-PCR 72 h after replating. Statistics shown by asterisks above the columns are relative to non-induced siCTRL transfected samples. * *p* < 0.05, ** *p* < 0.01, *** *p* < 0.001.

There have been conflicting observations on the effects of IL-6 and IL-1β on PCa cell proliferation [41,42]. In our experimental system, exposure to IL-6 or IL-1β alone for 72 h had no effect on C4-2B cell viability, whereas the presence of both cytokines in the medium significantly inhibited it (Figure 5a). This combinatorial effect was more prominent in the colony formation assay, which takes two weeks, where cotreatment with the two cytokines effectively and synergistically inhibited colony growth (Figure 5b,c). Interestingly, *STAMP2* knockdown increased C4-2B cell sensitivity to cytokine treatments. Both after three days of treatment in culture and in colony formation assays, *STAMP2* knockdown significantly increased cell loss relative to controls, both in response to single and double cytokine treatment (Figure 5a–d). This observation was further supported in *STAMP2* knockdown cells treated with cytokines by decreased expression of *MKI67* (proliferation marker protein Ki-67) and c-MYC, a well-established proto-oncogene, and increased expression of *CDKN1A* (cyclin-dependent kinase inhibitor 1/p21), which inhibits the cell cycle (Figure 5e). These data show that cytokine treatment inhibits PCa cell entry into the cell cycle, an effect that is exacerbated upon STAMP2 knockdown.

## 4. Discussion

Both in vitro and nude mice xenograft models showed that STAMP2 serves a critical role in PCa cell growth and survival [23]. Current data indicate that these activities are linked to the oxidoreductase activity of STAMP2 [20,21,22] which supports mitochondrial function [43], leading to an increase in reactive oxygen species (ROS) levels. ROS, in turn, activate expression of the transcription factor ATF4, a key stress response transcription factor that is essential for PCa growth [23,44]. Here, we show that STAMP2 expression is induced by inflammatory signaling in PCa cells and that STAMP2, in turn, regulates the impact of inflammatory cytokines on PCa cells.

Previous work has established that STAMP2 is an androgen-regulated gene [19]. Here, we found that STAMP2 is a direct AR target gene through three intronic AR-binding sites. Consistent with the very strong androgen induction of STAMP2, AR enrichment on one of these sites in the ChIP assay was comparable to that on the AR response element in the KLK3 enhancer region of the KLK3 gene, one of the canonical AR target genes. The cytokine regulation of STAMP2, however, is independent of its regulation by AR, and in fact is inhibited by it (see below). 

The inflammatory cytokines IL-6 and IL-1β significantly increased STAMP2 expression in LNCaP cells, as well as in its androgen-independent derivative, C4-2B cells. The STAMP2 induction profiles in response to single cytokine treatments were transient in serum-free culture conditions. This is similar to the physiological roles of STAMP2 in other tissues where it is transiently induced, for example, in the regulation of cellular responses to fluctuations in nutrients and inflammatory cues in adipocytes and in macrophages [24,30]. This dynamic and inducible regulation of STAMP2 is lost in obesity, a condition associated with chronically elevated levels of inflammatory stimuli [45]. Similarly, conditions of chronic inflammation may lead to dysregulated STAMP2 expression in PCa that would provide advantages to the cancer cell, e.g., through increased ROS levels, which may increase the rate of mutational events. 

Previous research suggested that paracrine inflammatory signaling cascades in the tumor microenvironment affect PCa hallmarks [4,5,6,7]. Among these, IL-6 and its intracellular mediator STAT3 have clear links to PCa progression, as they do with other cancer types [46,47,48]. IL-6/STAT3 signaling stimulates AR activity in PCa cells in the presence of androgens [49,50]. A constitutively active STAT3 mutant that is ectopically expressed in LNCaP cells enhanced AR recruitment to the PSA promoter [51]. Coimmunoprecipitation analyses of the cells ectopically expressing either AR or STAT3 indicated that they physically interact [52]; however, this has not been further explored in a more physiologically relevant setting. 

Consistent with these observations, we found that IL-6 cotreatment enhanced androgen-induced expression of STAMP2 in PCa cells. This suggested that cytokine treatment might further activate AR-mediated transcription of target genes, including STAMP2. Contrary to this hypothesis, however, both IL-6 and IL-1β induced STAMP2 expression independently of AR activity. In fact, perturbation of AR dramatically increased cytokine-induced STAMP2 expression. Androgen treatment also reduced expression of the STAT3 target gene SOCS3, while inhibition of AR activity exacerbated the expression of SOCS3 and the NF-κB target gene TNFAIP3. In addition, both basal and hormone-induced expression of canonical AR target genes, such as KLK3 and TMPRSS2, were consistently attenuated by IL-6 and IL-1β. Based on endogenous expression of these typical target genes as pathway reporters, there appears to be a negative two-way interaction between the androgen/AR pathway and both STAT3 and NF-κB signaling in these cells. Interestingly, this pathway crosstalk does not affect STAMP2 expression in the same way.

These two-way interactions between AR, STAT3, and NF-κB signaling pathways have previously been confirmed in different settings. Consistently, multiple studies have reported an inverse relationship between androgen levels and inflammatory signaling. For example, testosterone therapy relieved the symptoms of several chronic inflammatory diseases through inhibition of inflammatory cytokine expression and function [53]. In addition, cytokine treatment and NF-κB activation reduced AR activity in both LNCaP and C4-2B cells [37,54]. In fact, activation of the TNF/NF-κB axis reduced the global AR cistrome and androgen-induced transcriptome in LNCaP cells [55]. 

Similarly, AR perturbation increased STAT3 activity, both by siRNA-mediated knockdown in LNCaP cells [56] and by enzalutamide treatment in LNCaP, C4-2B, and 22Rv1 cell lines and xenografts [57]. Consistent with these findings, both IL-6R expression and STAT3 activation increase as PCa progresses, where a majority of CRPC bone metastases display elevated STAT3 activation [58]. Indeed, STAT3 activation has been suggested as a possible mechanism of resistance to androgen deprivation therapy through induction of epithelial-to-mesenchymal transition, increased chemokine synthesis, and macrophage recruitment [56]. Additional research is needed to identify the molecular mechanisms behind the crosstalk between AR and cytokine signaling pathways. In the case of STAMP2 regulation, AR inhibits the extent of cytokine induction, yet for IL-6, there are stimulatory effects when cells are treated with both IL-6 and androgens. This suggests that STAMP2 regulatory sequences and the factors that bind them have some unique features compared to other androgen- and cytokine-regulated genes that allow this complex regulation, which should be the topic of future studies.

Inflammatory signaling networks are rapidly inducible. The kinetics of cytokine-mediated STAMP2 expression differs to an extent from those observed for typical target genes of STAT3 and NF-κB. Transcription of IL-1β target genes begins within 30 min and can last for several hours [59], and is in contrast to significantly slower kinetics of STAMP2 induction. Induction of STAMP2 expression after IL-1β treatment might therefore be indirectly mediated by NF-κB, e.g., via expression of cell-autonomous or paracrine factors.

Opposed to single cytokine treatment, cotreatment of PCa cells with IL-6 and IL-1β resulted in a significant synergistic and more durable induction of STAMP2 expression. Both STAT3 and NF-κB were necessary for this synergistic regulation. Further studies of STAMP2 gene regulation in inflammatory conditions are needed to uncover the precise molecular mechanism(s) responsible for the synergistic effects induced by the two cytokines. 

It is currently not clear how STAMP2 may affect PCa cell proliferation under inflammatory stimuli. Prolonged exposure of LNCaP cells to IL-6, which might also chronically affect STAMP2 expression, results in a higher basal proliferation rate compared to control cells [60]. This suggests that LNCaP cells circumvent cytokine-induced growth-limiting mechanisms as a result of long-term conditioning. Both IL-6 and IL-1β attenuated the proliferation of cytokine-naïve C4-2B cells in the present study. The data further indicate that STAMP2 serves as an inducible survival factor in this in vitro inflammatory setting. STAMP2 knockdown cells fare worse than control cells in response to cytokine-mediated stress, which seems to involve effects on cell cycle progression. Future work is required to uncover whether the iron reductase activity of STAMP2 is required in this context, and further stress and survival pathways should be probed in order to identify those that are affected simultaneously by proinflammatory cytokines and STAMP2. More important for understanding PCa would be to determine how STAMP2 expression is affected by chronic inflammation in vivo, and whether STAMP2 plays an important role in the progression of inflammation-associated PCa.

## 5. Conclusions

The data presented here show for the first time that STAMP2, an androgen-regulated gene, may have a role in the inflammatory response of PCa cells. The inflammatory cytokines IL-6 and IL-1β markedly induce STAMP2 expression individually and in a synergistic manner. This does not require androgen/AR signaling, but depends on STAT3 and NF-kB pathways. In addition, inhibition of STAMP2 expression sensitized PCa cells to cytokine-induced growth arrest. Taken together, these results show that STAMP2 responds to inflammatory signaling and acts as a survival factor for AR-positive PCa cells under such conditions. Further research is needed to determine the exact molecular mechanisms of STAMP2 expression by STAT3 and NF-kB signaling crosstalk and how STAMP2 might operate as a survival factor in inflammatory conditions during prostate cancer progression.

## Data Availability

Not applicable.

## Supplementary Materials

The following are available online at https://www.mdpi.com/article/10.3390/cancers13071579/s1, Figure S1: Regulation of STAMP2 expression by cytokines; Figure S2: Cytokines negatively regulate AR signaling in PCa cells; Figure S3: Verification of small-molecule inhibitors.
