# Supplementary material for: STAMP2 Expression Mediated by Cytokines Attenuates Their Growth-Limiting Effects in Prostate Cancer Cells
Source: Cancers (Basel). 2021 Mar 30;13(7):1579. doi: 10.3390/cancers13071579 (PMC8036285; doi:10.3390/cancers13071579)
Supplement: Supplementary file 1 [file cancers-13-01579-s001.pdf]

Supplementary Figure 1

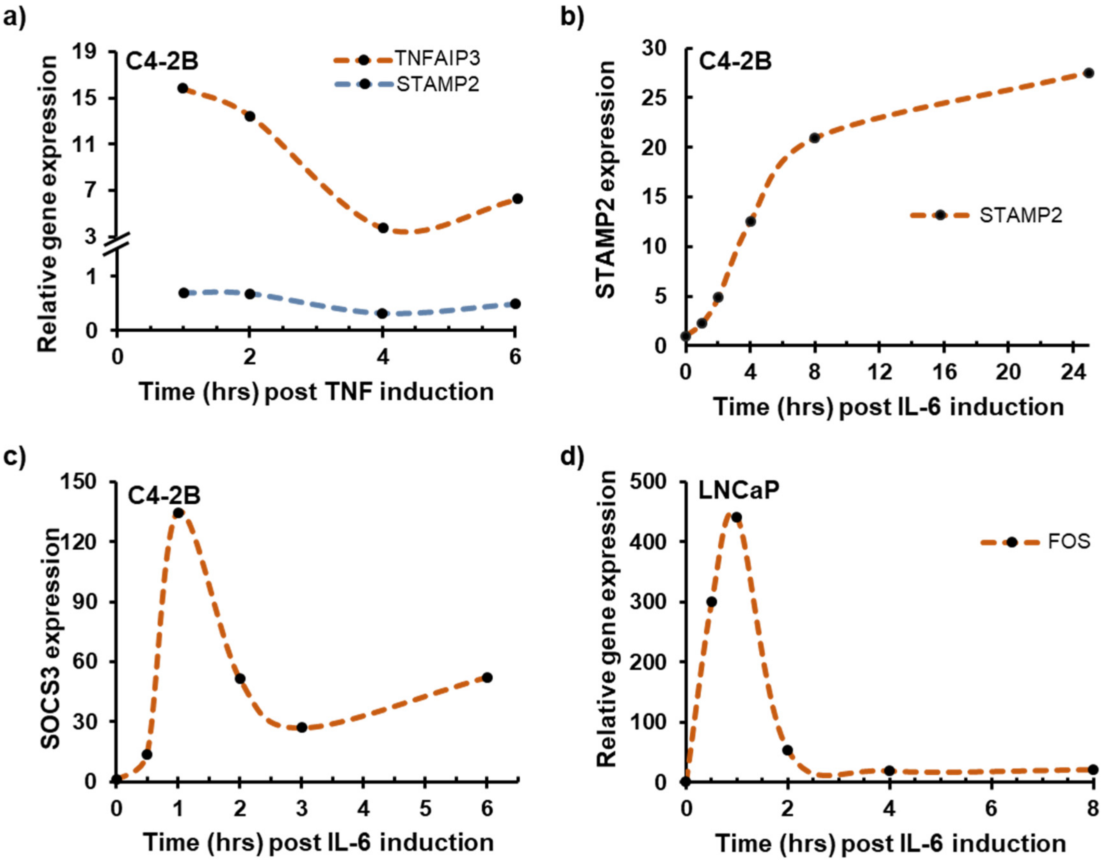

Supplementary Figure 1: Regulation of STAMP2 expression by cytokines. a-d) Cells were serum-starved before induction with 20 ng/mL TNF **(a)** or IL-6 **(b-d)**. Total RNA was harvested at the indicated time points and subjected to qRT-PCR analysis.

Supplementary Figure 2

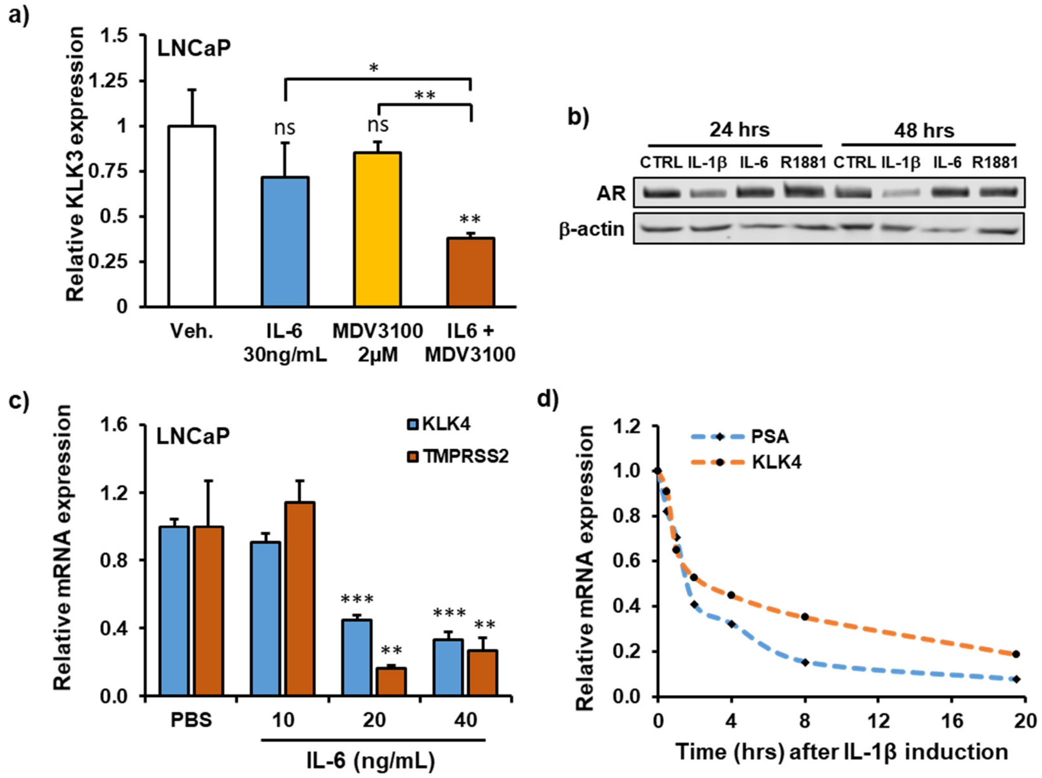

Supplementary Figure 2: Cytokines negatively regulate AR signaling in PCa cells. a) Serum-starved LNCaP cells were pretreated with or without enzalutamide (MDV) for 2 hours and then IL-6 treatment was performed. Total RNA was harvested 16 hours later and subjected to qRT-PCR analysis to detect KLK3 expression levels. Statistics shown by asterisks above the columns are relative to vehicle treatment. **b)** LNCaP cells were treated with 20 ng/ml IL-1β or IL-6, or 1 nM R1881. At indicated time points, cells were harvested and Western blotting was performed to detect AR levels. **c-d)** LNCaP cells were serum-starved before induction with increasing concentrations of IL-6 (for 16 hours) **(c)** or 20 ng/mL IL-1β **(d)**. Total RNA was harvested and expression of the KLK4, TMPRSS2, and PSA were detected by qRT-PCR. Statistics shown by asterisks above the columns are relative to PBS treatment.

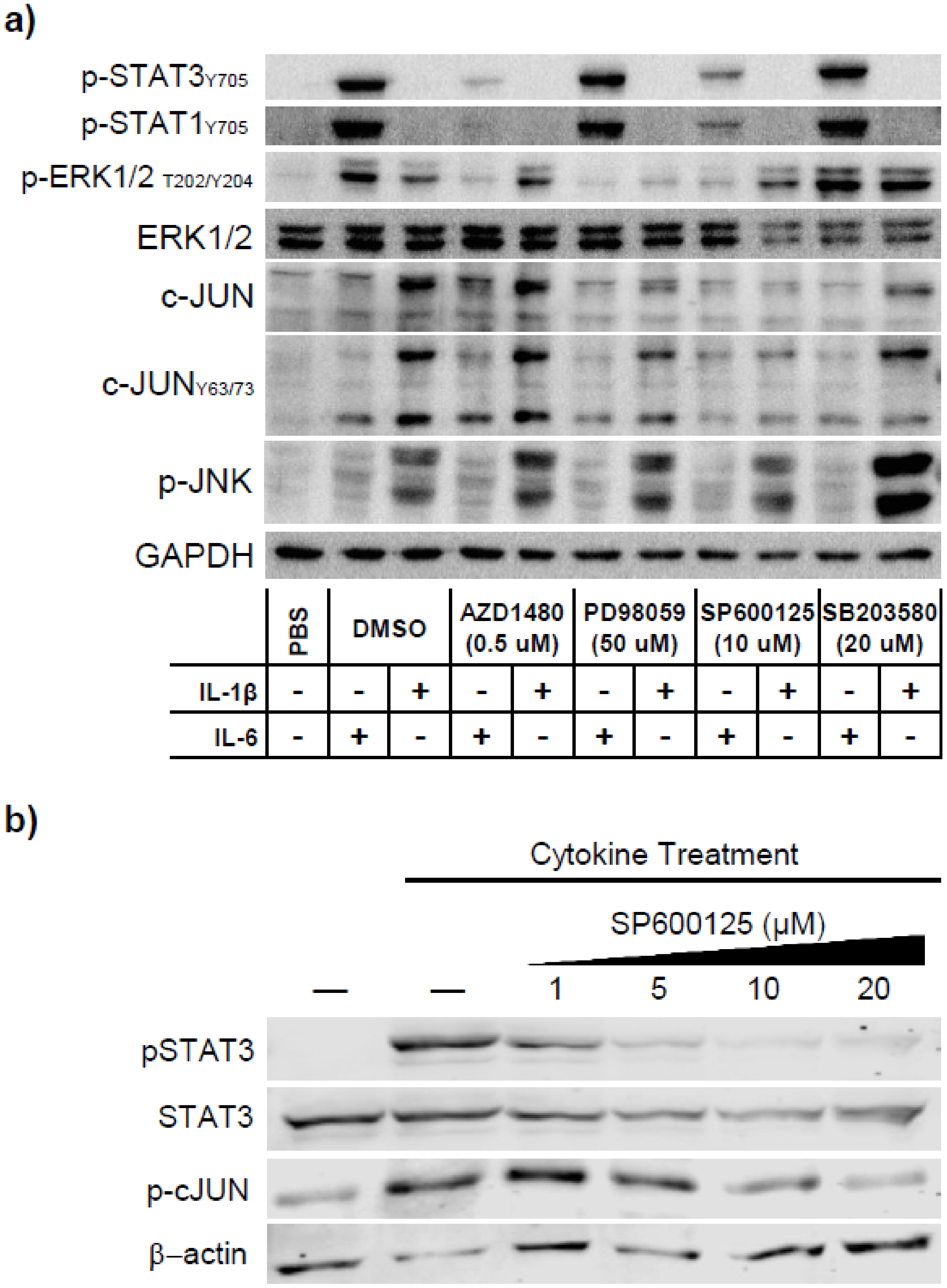

Supplementary Figure3:Verification of the small molecule inhibitors.a) To validate the activity of small molecule inhibitors(AZD1480–JAK1/2/3inhibitor;PD98059–MEK1/2inhibitor;SP600125–JNKinhibitor;SB203580p38MAPKinhibitor)LNCaP cells were treated with the indicated compounds for two hours and induced with 20ng/mLIL6 or IL1B for 35min.Lysates were

harvested and Western blotting was performed.**b)**C42B cells were treated with increasing concentrations of SP600125 for 4 hours and induced with 20ng/mlIL 1b/IL6 combination.45 min after induction with cytokines,cells were harvested and Western blotting was performed.

UNCROPPED WESTERN BLOT SCANS

Figure 3C

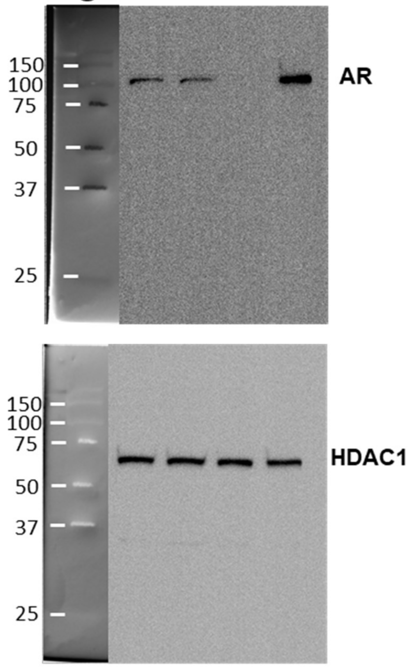

Figure 3E

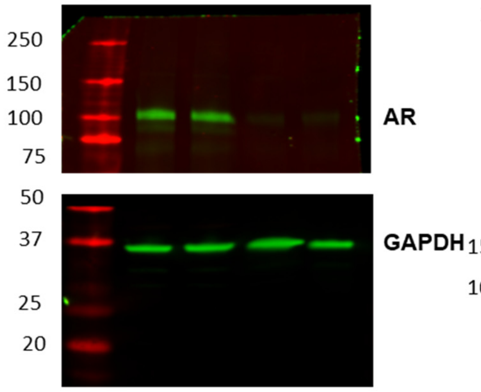

Figure 4D

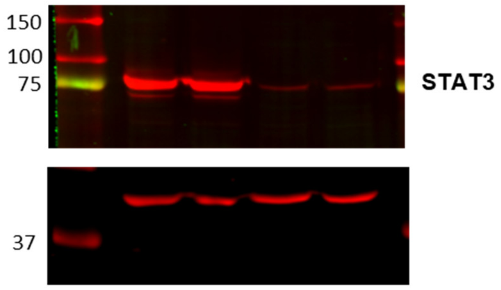

Figure 4E

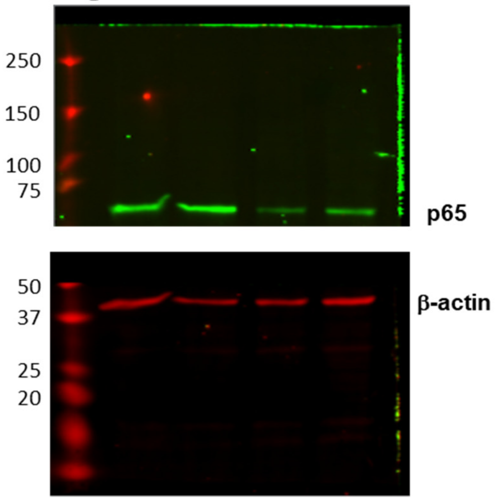

Figure S2B

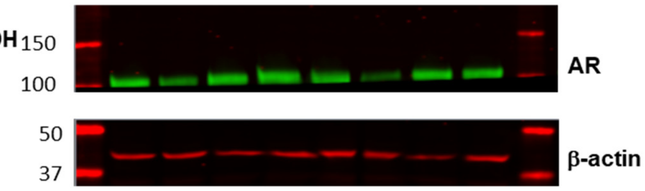

UNCROPPED WESTERN BLOT SCANS

Figure 2D

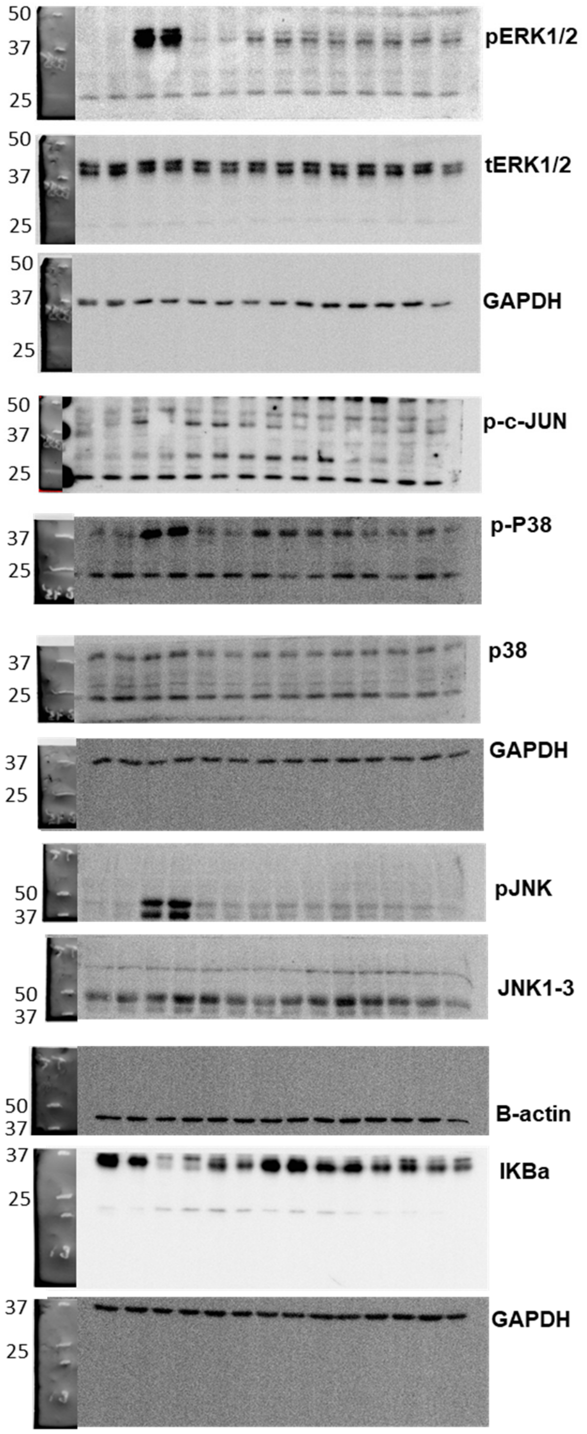

Figure 2I

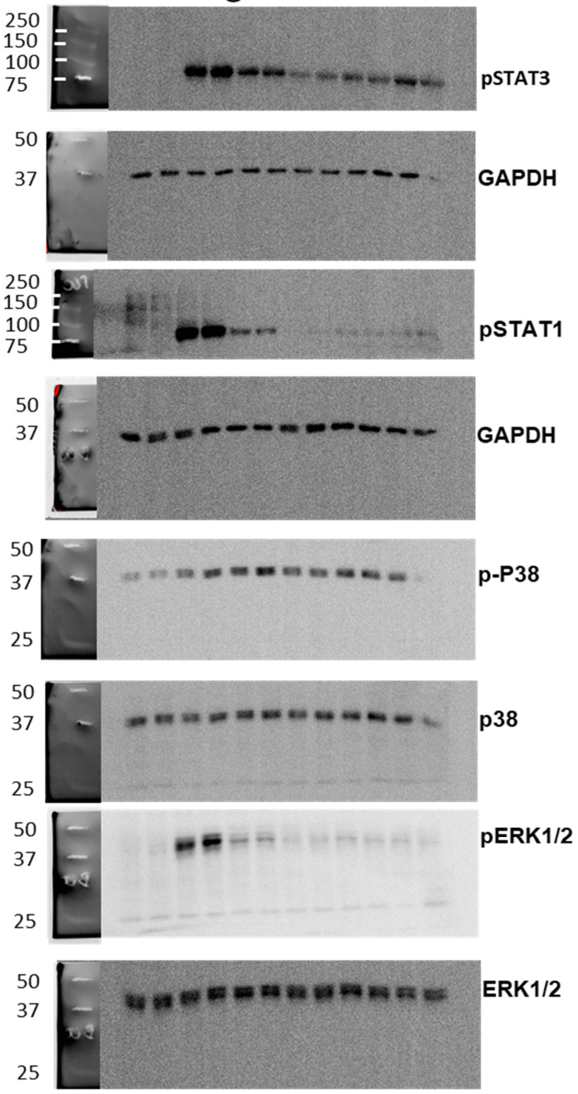

UNCROPPED WESTERN BLOT SCANS

Figure S3A

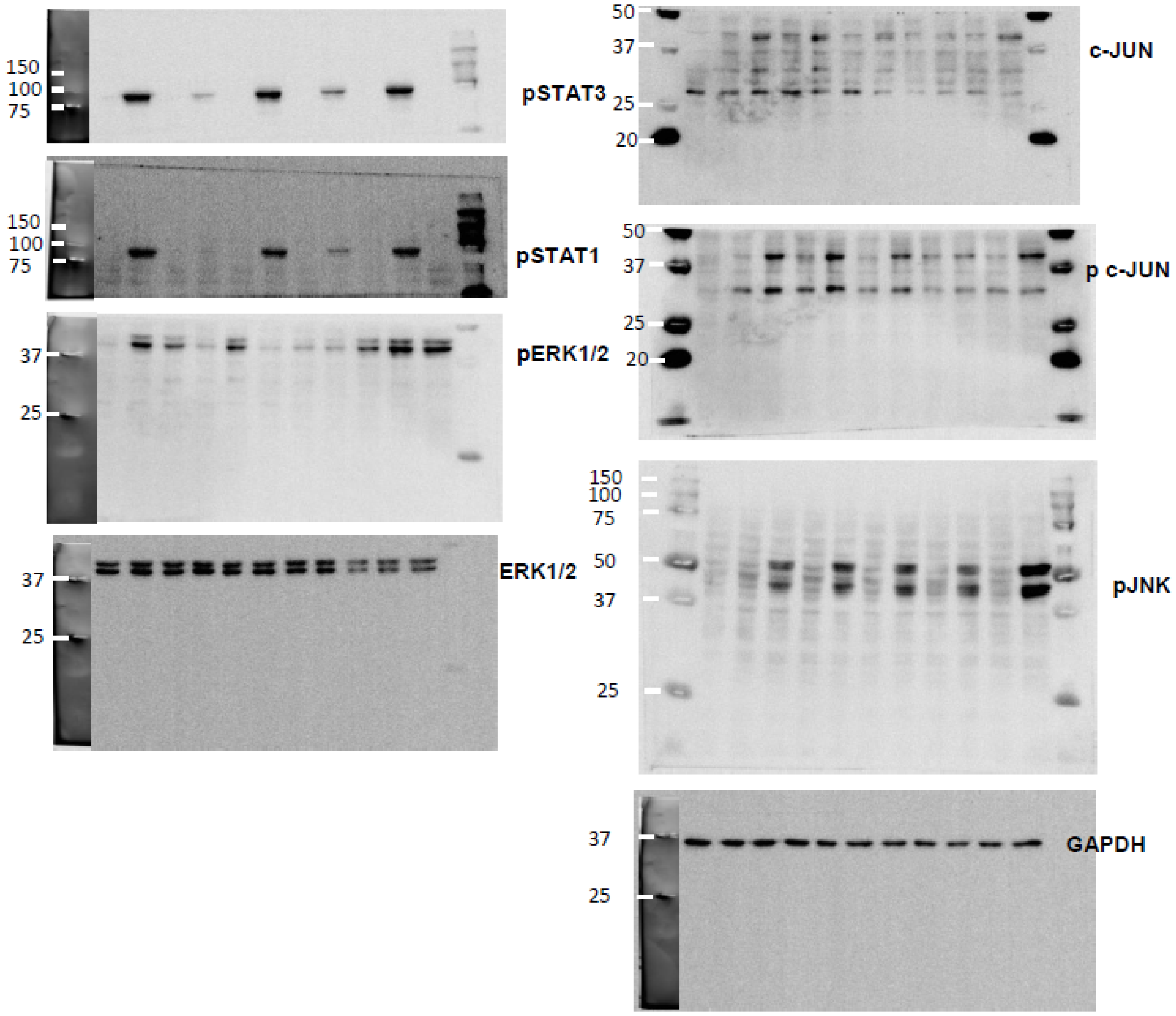

QUANTIFICATION OF WESTERN BLOTS

Figure 2D

Lane #	pERK1/2	ERK1/2	GAPDH	p-cJUN	p-p38	p38	GAPDH	pJNK1/3	JNK1/3	B-actin	IKBA	GAPDH
1	688.7	7625.4	7443.2	5733.6	2538.1	3716.0	5600.9	1263.1	3747.2	6544.5	9736.2	5168.3
2	733.2	8302.8	7202.4	4841.1	2754.8	3827.0	6652.1	1066.4	5625.1	5959.4	7232.8	5540.1
3	10130.1	8039.8	6105.6	7626.0	6742.3	4241.8	4128.3	8514.8	6268.5	4901.8	2116.6	4213.1
4	9364.1	7323.8	5331.9	1203.5	7416.5	4850.8	5578.7	10428.2	8932.6	6177.2	3968.9	4651.7
5	976.9	6479.3	5602.5	6000.6	3719.2	3102.5	5204.1	1849.7	7130.9	6034.5	6597.9	5215.5
6	1093.0	5544.4	5339.2	5962.1	2462.2	2407.8	4059.9	1183.3	5666.9	5242.8	4615.6	4004.6
7	2552.7	6376.7	4469.7	4050.4	4768.9	4079.9	4652.3	1406.7	4795.1	5286.1	8849.2	3869.9
8	2847.5	6664.6	5018.1	3742.7	4221.9	3557.9	5142.9	1557.7	5564.1	5471.9	9427.4	3573.8
9	2557.5	6941.0	6971.4	2213.1	3220.8	2826.2	5158.1	1319.6	6132.4	5564.5	7178.6	4469.5
10	2513.2	6931.1	7150.2	2427.8	4220.1	4386.3	7258.7	2225.8	9248.2	6406.9	7427.1	3795.1
11	2716.7	7556.7	7028.5	2341.5	2597.0	3221.9	5406.6	2017.9	7482.5	6252.4	5505.8	4092.1
12	2763.7	6802.4	6615.2	4496.0	3365.4	3960.5	5326.8	1715.8	5830.9	5838.6	6664.1	3631.3
13	2729.5	7025.2	7570.5	5148.1	3962.8	4255.6	5715.5	1505.8	5213.2	6258.8	4876.8	4486.4
14	2424.1	4250.5	4180.4	5208.7	1768.8	1537.0	4231.8	670.7	2259.4	3312.2	4148.2	2708.5

Figure 2I

Lane #	pSTAT3	GAPDH	pSTAT1	GAPDH	pP38	p38	GAPDH	pERK1/2	ERK1/2	GAPDH
1	192.7	6188.7	807.2	8190.0	929.8	3897.9	6188.7	741.6	3377.2	8190.0
2	152.3	6078.3	943.9	7864.9	1295.5	4330.8	6078.3	940.5	4027.5	7864.9
3	9814.1	4869.4	10691.4	8660.7	2623.8	3544.6	4869.4	8708.0	4370.2	8660.7
4	10105.4	5482.3	10656.5	7596.4	3805.9	3814.6	5482.3	9532.2	5254.6	7596.4
5	4328.6	5729.7	2050.3	7060.9	3730.6	4056.9	5729.7	2690.1	5624.4	7060.9
6	4119.2	5103.1	1876.7	6620.9	5143.7	4301.5	5103.1	2142.6	5386.7	6620.9
7	1003.8	5102.6	689.3	6910.6	3210.1	3517.1	5102.6	776.8	4951.0	6910.6
8	1799.2	5674.4	810.4	10047.0	3030.6	4153.4	5674.4	1019.2	5764.0	10047.0
9	2845.3	6308.4	567.4	9674.6	3955.6	4402.5	6308.4	1388.4	6205.5	9674.6
10	2470.3	7803.7	459.6	8599.1	3799.6	5069.6	7803.7	1230.5	5092.3	8599.1
11	4828.9	7666.1	605.3	6919.2	3173.3	4439.0	7666.1	1313.9	3929.2	6919.2
12	2858.9	1393.2	1304.4	5215.7	209.7	1521.9	1393.2	1042.5	4005.1	5215.7

Figure 3C

Lane #	AR	HDAC1
1	6402.3	16316.0
2	7395.3	19548.1
3	1652.7	17683.0
4	20386.9	16516.7

Figure 3E

Lane #	AR	GAPDH
1	23273.0	11678.8
2	22514.2	12507.2
3	2263.5	15103.3
4	2562.8	9550.2

Figure 4D

Lane #	STAT3	B-actin
1	27509.5	18705.4
2	26695.3	11297.6
3	1474.4	19290.1
4	1789.6	16773.8

Figure 4E

Lane #	p65	B-actin
1	27224.3	25463.5
2	32867.1	14134.1
3	7769.7	18253.1
4	12807.3	30114.7

Figure S2

Lane #	AR	B-actin
1	4808.1	2913.2
2	2474.3	2899.0
3	5573.9	2047.4
4	6839.0	2990.7
5	4574.5	3225.1
6	1419.6	2499.5
7	5215.2	1423.5
8	4921.8	3176.4
